# 24-Month Efficacy and Safety Results from Japanese Patients in the IMPERIAL Randomized Study of the Eluvia Drug-Eluting Stent and the Zilver PTX Drug-Coated Stent

**DOI:** 10.1007/s00270-021-02901-6

**Published:** 2021-07-07

**Authors:** Osamu Iida, Masahiko Fujihara, Daizo Kawasaki, Shinsuke Mori, Hiroyoshi Yokoi, Akira Miyamoto, Kimihiko Kichikawa, Masato Nakamura, Takao Ohki, Juan Diaz-Cartelle, Stefan Müller-Hülsbeck, William A. Gray, Yoshimitsu Soga

**Affiliations:** 1 Kansai Rosai Hospital, Amagasaki, Hyogo Japan; 2 Kishiwada Tokushukai Hospital, Osaka, Japan; 3 Morinomiya Hospital, Osaka, Japan; 4 Saiseikai Yokohama-City Eastern Hospital, Yokohama, Japan; 5 Fukuoka Sanno Hospital, Fukuoka, Japan; 6 Takatsu General Hospital, Kawasaki, Japan; 7 Nara Medical University Hospital, Kashihara, Japan; 8 Toho University Ohashi Medical Center, Tokyo, Japan; 9 The Jikei University Hospital, Tokyo, Japan; 10 Boston Scientific, Marlborough, MA USA; 11 DIAKO Krankenhaus GmbH Flensburg, Flensburg, Germany; 12 Lankenau Heart Institute, Wynnewood, PA USA; 13 Kokura Memorial Hospital, Kitakyushu, Japan

**Keywords:** Drug-eluting stent, Paclitaxel, Peripheral arterial disease, Superficial femoral artery, Vascular patency

## Abstract

**Purpose:**

The purpose of the study is to report 24-month efficacy and safety results for the Japanese patient cohort in a prospective randomized controlled trial (RCT) of drug-eluting stent (DES) use for peripheral artery disease.

**Materials and methods:**

Patients in the global IMPERIAL RCT had femoropopliteal lesions treated with either the Eluvia DES (Boston Scientific, Marlborough, MA, USA) or the Zilver PTX drug-coated stent (Cook Medical, Bloomington, IN, USA). At 24 months, assessments included duplex ultrasound imaging for core laboratory vessel patency measurement, target lesion revascularization (TLR) rates, and clinical outcome measures.

**Results:**

The Japanese cohort included 84 patients (56 treated with Eluvia and 28 with Zilver PTX). The clinically driven TLR rates were 5.6% (3/54) and 18.5% (5/27) for patients treated with Eluvia and Zilver PTX, respectively (difference -13.0%, 95%CI -28.8, 2.9%; *p* = 0.11). The Kaplan–Meier estimates for freedom from clinically driven TLR at 24 months were 94.3% for patients who received Eluvia and 80.4% for those who received Zilver PTX (log rank *p* = 0.05), and for primary patency they were 88.5% and 80.4%, respectively (log rank *p* = 0.28). Mortality rates were 5.6% (3/54) and 11.1% (3/27); *p* = 0.39. Rutherford classification improved by at least one category without TLR for 91.8% (45/49) and 68.2% (15/22) of patients (*p *= 0.03). Walking impairment score improvements were sustained over time.

**Conclusion:**

The results at 24 months support the efficacy and safety of DES in Japanese patients, with sustained clinical improvements and numerically fewer reinterventions for those treated with Eluvia.

**Clinical trial Registration:**

Clinicaltrials.gov identifier NCT02574481.https://clinicaltrials.gov/ct2/show/NCT02574481

**Level of Evidence:**

EBM Level III; cohort analysis of randomized trial.

## Introduction

The ability of drug-eluting endovascular treatment of femoropopliteal disease to reduce the need for reinterventions has been well described, particularly in the short term and for Caucasian populations [1–6]. Longer-term prospective data are important to characterize the outcomes expected for patients over time [7–10], and large randomized studies provide opportunity to explore clinical outcomes over time for various patient cohorts [11].

The IMPERIAL randomized controlled study [10, 12] is a comparison of the durable-polymer-coated Eluvia DES (Boston Scientific, Marlborough, MA, USA) and polymer-free Zilver PTX drug-coated stent (Cook Corporation, Bloomington, IN, USA). Among global randomized patients, Eluvia DES demonstrated superior efficacy at 1 year [12] with favorable outcomes sustained at 2 years [10].

Peripheral artery disease (PAD) is highly prevalent in Japan [13], and identifying effective treatments is important in this population. One-year results in the cohort of patients from Japan showed outstanding patency and safety [14], and we sought to determine whether the 2-year outcomes for Japanese patients were likewise reflective of the overall trial findings. This report presents the primary patency, freedom from clinically driven target lesion revascularization (CD-TLR), patient outcome assessments, and safety for Japanese patients in the IMPERIAL randomized trial through 24 months.

## Materials and Methods

### Study Population

IMPERIAL randomized trial methods were reported previously [12]. Eligible patients had stenotic or occlusive femoropopliteal artery lesions (total length 30–140 mm) and Rutherford category 2, 3, or 4 symptoms at presentation. Informed consent was required from all study patients. Patients were randomly assigned to treatment with either Eluvia DES or Zilver PTX in a 2:1 ratio. Of the 465 patients in the global randomized trial, 84 were enrolled across 10 Japanese sites (*n* = 56 Eluvia, *n* = 28 Zilver PTX; 18% of the overall study sample).

### Assessments and Definitions

Scheduled assessments at 24 months (730 ± 30 days) post-procedure included the following: duplex ultrasound (DUS) imaging for vessel patency measurement by the ultrasound core laboratory (VasCore, Boston, MA, USA), Rutherford category for clinical improvement, ankle-brachial index (ABI), the Walking Impairment Questionnaire (WIQ), EQ-5D health-related quality of life assessment, and antiplatelet medication use. Japanese translations of the WIQ and EQ-5D were administered at Japanese sites. An independent Clinical Events Committee adjudicated TLR, target limb amputation, stent thrombosis, and death reported through 24-month follow-up.

CD-TLR was defined as a reintervention within 5 mm proximal or distal to the original treatment segment for angiographic diameter stenosis ≥ 50% in the presence of recurrent symptoms (i.e., increase in Rutherford class by 1 or more) or ankle-brachial index decrease in at least 0.15 or 20% compared with post-treatment in the treated segment. Primary patency was defined for target stented segments as core-lab evaluated peak systolic velocity ratio ≤ 2.4 and without CD-TLR or bypass of the target lesion.

### Statistical Analysis

These cohort analyses were not powered for hypothesis testing and thus are considered exploratory. Event rates are reported for safety measures adjudicated by the Clinical Events Committee, and 95% confidence intervals were calculated around the difference between intervention groups. Statistical significance testing of categorical variables was performed with two-sided Fisher’s exact test or Chi-square test. For continuous variables, *p*-values are from 2-sided *t*-tests. Kaplan–Meier curves for freedom from CD-TLR and primary patency were generated with standard errors; log rank *p*-values were calculated. Kaplan–Meier patency estimates were based on the time to event of CD-TLR up to 730 days and duplex ultrasound data at 24 months. Statistical analyses were performed with Statistical Analysis Software, version 9.2 or later (SAS Institute Inc., Cary, North Carolina, USA).

## Results

### Patients

Baseline characteristics of the 84 Japanese patients enrolled in IMPERIAL are summarized in Table 1. Mean lesion length was 91.8 ± 38.0 mm for patients treated with Eluvia and 87.4 ± 41.7 mm for patients treated with Zilver PTX. Moderate or severe calcification was present in 60.7% and 82.1% of patients, respectively. Seventy-one patients completed the 24-month visit, and 3 patients in each treatment arm died prior to the visit.

**Table 1:** Baseline characteristics of IMPERIAL patients enrolled in Japan

	Eluvia (*N* = 56)	Zilver PTX (*N* = 28)	*p*
Age (y)	73.7 ± 7.7	74.3 ± 7.3	0.71
Male	76.8% (43/56)	75.0% (21/28)	0.86
Smoking history			
Current	16.1% (9/56)	25.0% (7/28)	0.33
Previous	62.5% (35/56)	60.7% (17/28)	0.87
Medically treated diabetes mellitus	53.6% (30/56)	53.6% (15/28)	1.00
ABI	0.7 ± 0.2	0.7 ± 0.2	1.00
Arterial Segments			
Ostial	1.8% (1/56)	0.0% (0/28)	1.00^a^
Proximal superficial femoral artery	17.9% (10/56)	14.3% (4/28)	0.77^a^
Mid-superficial femoral artery	71.4% (40/56)	71.4% (20/28)	1.00
Distal superficial femoral artery or proximal popliteal artery	50.0% (28/56)	60.7% (17/28)	0.35
Lesion length (mm)	91.8 ± 38.0	87.4 ± 41.7	0.63
Reference vessel diameter (mm)	5.1 ± 0.8	5.0 ± 0.7	0.49
Calcification			
None/mild	39.3% (22/56)	17.9% (5/28)	0.05
Moderate	32.1% (18/56)	50.0% (14/28)	0.11
Severe	28.6% (16/56)	32.1% (9/28)	0.74
% Diameter stenosis	79.9 ± 14.0	74.9 ± 14.3	0.12
50%-99%	80.4% (45/56)	82.1% (23/28)	0.84
100% (Occlusion)	19.6% (11/56)	17.9% (5/28)	0.84

Continuous data are presented as the means  ±  standard deviation; categorical data are given as the percentage (count). All lesion characteristics are as reported by the angiographic core laboratory

^a^*p*-values from 2-sided Fisher’s exact test

### Safety and Efficacy

**Fig. 1:**
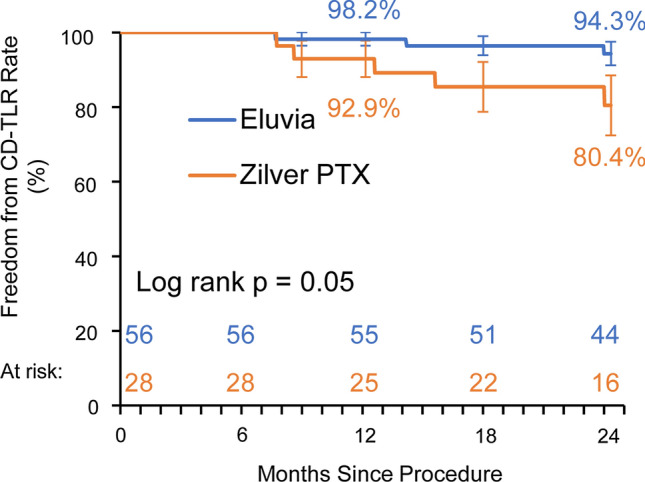
Kaplan–Meier estimate of freedom from CD-TLR and standard errors

**Fig. 2:**
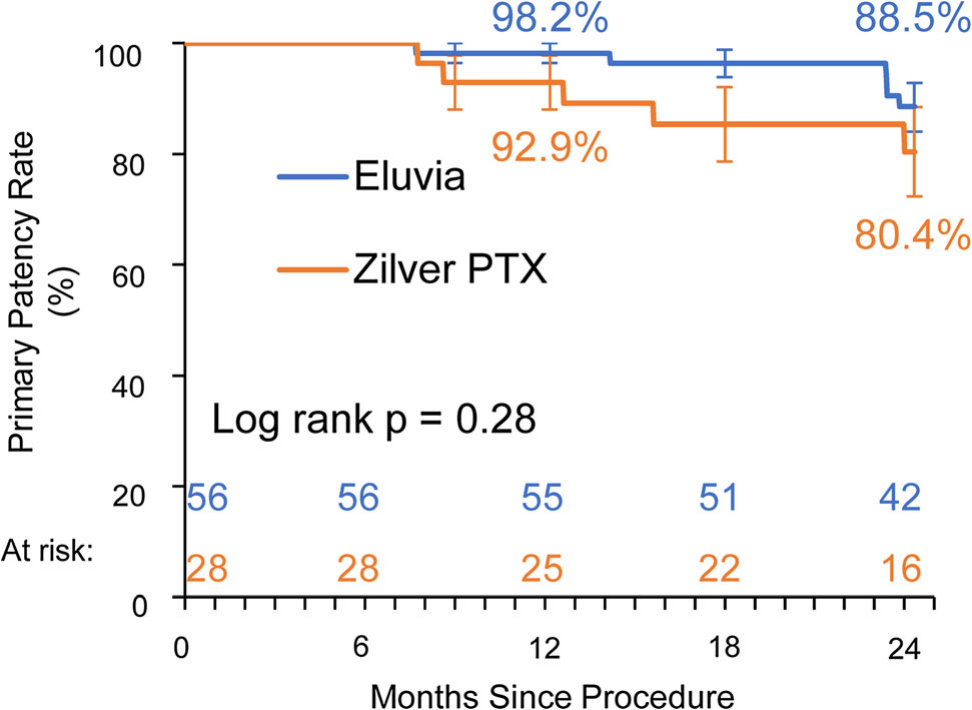
Kaplan–Meier estimate of primary patency and standard errors

Events adjudicated by the Clinical Events Committee are shown in Table 2. As shown in the table, the 24-month CD-TLR rate for patients treated with Eluvia was less than one-third that of patients treated with Zilver PTX. Kaplan–Meier estimates of freedom from CD-TLR and primary patency are shown in Figs. 1 and 2, respectively. The Kaplan–Meier estimate for freedom from CD-TLR at 24 months was 94.3% for patients who received Eluvia and 80.4% for those who received Zilver PTX (log rank *p* = 0.05; Fig. 1).

**Table 2:** Events adjudicated by the Clinical Events Committee through 24 months^a^

	Eluvia (*n* = 56)	Zilver PTX (*n* = 28)	Difference [95% CI]	*p*^b^
All deaths	5.6% (3/54)	11.1% (3/27)	− 5.6% [− 18.9%, 7.8%]	0.39
Target lesion revascularization^c^	5.6% (3/54)	18.5% (5/27)	− 13.0% [− 28.8%, 2.9%]	0.11
Target limb amputation	0.0% (0/54)	3.7% (1/27)	− 3.7% [− 10.8%, 3.4%]	0.33
Stent thrombosis	1.9% (1/54)	0.0% (0/27)	1.9% [− 1.7%, 5.4%]	1.00

^a^The CEC-adjudicated denominator is based on 1) subjects with CEC-adjudicated events (i.e., any death, target lesion/vessel revascularization, target limb amputation, stent thrombosis) through 24 months and 2) subjects with no events but their follow-up time reach on (or beyond) the earliest visit window

^b^P values from 2-sided Fisher’s exact test

^c^All target lesion revascularizations met the criteria for “clinically driven;” i.e., a reintervention within 5 mm proximal or distal to the original treatment segment for angiographic diameter stenosis  ≥ 50% in the presence of recurrent symptoms (i.e., increase in Rutherford class by 1 or more) or ABI decrease of at least 0.15 or 20% in the treated segment

Kaplan–Meier estimates of primary patency at 24 months were 88.5% and 80.4% for Eluvia and Zilver PTX-treated patients, respectively (log rank *p* = 0.28; Fig. 2).

No new stent thrombosis was reported between 12- and 24-month follow-up. One patient in the Eluvia arm had stent thrombosis (Table 2), and TLR reported prior to 12-month follow-up as described previously [14]. DUS imaging at the patient’s 24-month visit revealed stenosis proximal to the stent and stent occlusion.

All but one Japanese patient (Eluvia arm) who completed the 24-month visit had diagnostic radiography performed to examine stent integrity, and no new fractures were observed. One fracture had been identified in the Eluvia arm at the 12-month visit [14], and the stent was confirmed patent at 24 months; no associated complications or adverse events were reported.

### Antiplatelet Medication Use

The study protocol required dual antiplatelet therapy for at least the first 60 days post-procedure. Use diminished over time with 24-month rates of 49.0% (24/49) and 40.9% (9/22) for Eluvia and Zilver PTX, respectively, reported for dual antiplatelet therapy (*p* = 0.53). Antiplatelet monotherapy was recommended to continue through trial completion. At 24 months, acetylsalicylic acid use was reported by 75.5% (37/49) of Eluvia patients and 77.3% (17/22) of Zilver PTX patients (*p* = 0.87).

### Clinical Outcomes

**Fig. 3:**
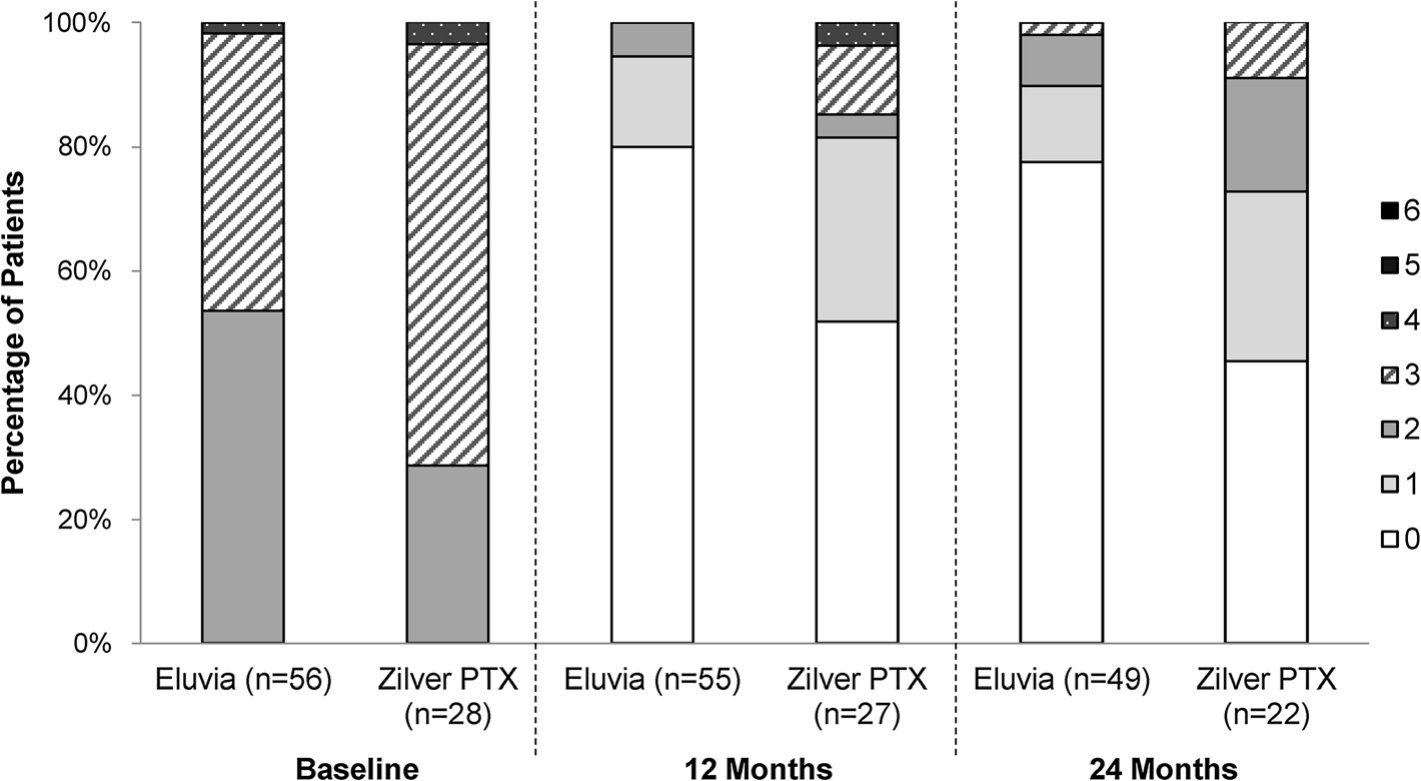
Rutherford category distribution

The Rutherford category distribution is shown in Fig. 3. At 24 months, 89.8% (44/49) of patients treated with Eluvia and 72.7% (16/22) of patients treated with Zilver PTX presented with symptoms categorized as 0 or 1, with primary sustained clinical improvement (i.e., improvement in Rutherford classification by one or more categories as compared with baseline and without TLR) for 91.8% (45/49) of patients treated with Eluvia and 68.2% (15/22) of patients treated with Zilver PTX (*p* = 0.03).

ABI improvement was sustained through 24 months, with mean ± SD values of 0.9 ± 0.2 and 0.9 ± 0.1 for patients treated with Eluvia and Zilver PTX, respectively. A total of 78.0% (39/50) of patients treated with Eluvia and 65.2% (15/23) of patients treated with Zilver PTX showed hemodynamic improvement (*p* = 0.25), defined as ABI increase ≥ 0.10 from baseline or achievement of ABI value ≥ 0.90 without TLR.

Japanese patients in the study had sustained improvements in walking impairment scores over time (Table 3). The health-related quality of life measure showed that the distribution of patients experiencing problems with mobility or pain/discomfort shifted toward less severity (Online Resource 1). At 24 months, 40.8% and 27.3% of Eluvia- and Zilver PTX-treated patients (*p* = 0.27), respectively, showed improvement in mobility compared with baseline, and improvement in the pain/discomfort dimension was reported by 38.8% and 40.9% (*p* = 0.86), respectively (Table 4).

**Table 3:** Walking Impairment Questionnaire

	Baseline	1 Month	6 Months	12 Months	24 Months
Eluvia					
Walking impairment	58.48 ± 32.78 (56)	92.41 ± 17.79 (56)	90.63 ± 15.49 (56)	84.55 ± 21.78 (55)	77.55 ± 26.64 (49)
Change from baseline	–	33.93 ± 34.19 (56)	32.14 ±34.28 (56)	25.91 ± 39.95 (55)	21.43 ± 38.19 (49)
*P*^a^	–	< .0001	< .0001	< .0001	0.0003
Distance	58.10 ± 32.16 (56)	92.14 ± 18.54 (56)	87.96 ± 24.12 (56)	84.84 ± 28.98 (55)	80.54 ± 33.33 (49)
Change from baseline	–	34.04 ± 30.12 (56)	29.86 ± 29.46 (56)	27.51 ± 32.10 (55)	23.16 ± 34.65 (49)
*P*^a^	–	< .0001	< .0001	< .0001	< .0001
Speed	39.62 ± 21.01 (56)	52.23 ± 25.54 (56)	50.87 ± 24.27 (56)	51.42 ± 26.18 (55)	51.71 ± 31.13 (49)
Change from baseline	–	12.62 ± 22.34 (56)	11.26 ± 23.85 (56)	11.56 ± 25.35 (55)	12.91 ± 26.44 (49)
*P*^a^	–	< .0001	0.0008	0.0013	0.0013
Stair climbing	56.85 ± 32.73 (56)	74.26 ± 29.17 (56)	73.44 ± 30.09 (56)	67.12 ± 33.30 (55)	72.96 ± 34.23 (49)
Change from baseline	–	17.41 ± 29.09 (56)	16.59 ± 31.73 (56)	9.54 ± 30.55 (55)	14.88 ± 33.07 (49)
*P*^a^	–	< .0001	0.0003	0.0243	0.0028
Zilver PTX					
Walking impairment	58.04 ± 25.51 (28)	84.82 ± 23.90 (28)	73.21 ± 32.58 (28)	71.30 ± 33.76 (27)	75.00 ± 29.88 (22)
Change from baseline	–	26.79 ± 28.81 (28)	15.18 ± 34.25 (28)	12.96 ± 35.61 (27)	17.05 ± 30.26 (22)
*P*^a^	–	< .0001	0.0266	0.0697	0.0152
Distance	57.68 ± 33.84 (28)	80.18 ± 24.71 (28)	71.40 ± 33.91 (28)	64.33 ± 37.01 (27)	67.97 ± 39.93 (22)
Change from baseline	–	22.50 ± 29.02 (28)	13.72 ± 27.84 (28)	4.67 ± 31.61 (27)	5.69 ± 29.78 (22)
*P*^a^	–	0.0003	0.0147	0.4501	0.3803
Speed	37.38 ± 21.36 (28)	48.10 ± 24.36 (28)	44.53 ± 27.46 (28)	43.20 ± 31.52 (27)	46.39 ± 30.19 (22)
Change from baseline	–	10.71 ± 19.57 (28)	7.14 ± 21.90 (28)	5.23 ± 26.34 (27)	5.88 ± 27.36 (22)
*P*^a^	–	0.0074	0.0958	0.3114	0.3249
Stair climbing	44.20 ± 33.15 (28)	56.10 ± 35.49 (28)	55.66 ± 37.03 (28)	57.10 ± 40.94 (27)	63.26 ± 40.14 (22)
Change from baseline	–	11.90 ± 30.00 (28)	11.46 ± 39.29 (28)	13.58 ± 37.22 (27)	18.56 ± 35.21 (22)
*P*^a^	–	0.0452	0.1345	0.0692	0.0220

Values are mean ± SD (n)

^a^*P*-values calculated by paired t-test

**Table 4:** Improvement in EQ-5D health-related quality of life dimensions

	Eluvia	Zilver PTX	Difference [95% CI]	*p*
1 Month				
Mobility	48.2% (27/56)	35.7% (10/28)	12.5% [− 9.6%, 34.6%]	0.28
Self-care	1.8% (1/56)	3.6% (1/28)	− 1.8% [− 9.5%, 5.9%]	1.00^a^
Usual activities	19.6% (11/56)	35.7% (10/28)	− 16.1% [− 36.6%, 4.5%]	0.11
Pain/discomfort	44.6% (25/56)	39.3% (11/28)	5.4% [− 16.9%, 27.6%]	0.64
Anxiety/depression	10.7% (6/56)	17.9% (5/28)	− 7.1% [− 23.5%, 9.2%]	0.49^a^
6 Months				
Mobility	48.2% (27/56)	21.4% (6/28)	26.8% [6.7%, 46.8%]	0.02
Self-care	1.8% (1/56)	0.0% (0/28)	1.8% [− 1.7%, 5.3%]	1.00^a^
Usual activities	19.6% (11/56)	32.1% (9/28)	− 12.5% [− 32.7%, 7.7%]	0.20
Pain/discomfort	41.1% (23/56)	28.6% (8/28)	12.5% [− 8.6%, 33.6%]	0.26
Anxiety/depression	8.9% (5/56)	21.4% (6/28)	− 12.5% [− 29.4%, 4.4%]	0.17^a^
12 Months				
Mobility	40.0% (22/55)	22.2% (6/27)	17.8% [− 2.6%, 38.1%]	0.11
Self-care	1.8% (1/55)	0.0% (0/27)	1.8% [− 1.7%, 5.3%]	1.00^a^
Usual activities	20.0% (11/55)	33.3% (9/27)	− 13.3% [− 34.0%, 7.4%]	0.19
Pain/discomfort	47.3% (26/55)	25.9% (7/27)	21.3% [0.2%, 42.5%]	0.06
Anxiety/depression	10.9% (6/55)	11.1% (3/27)	− 0.2% [− 14.6%, 14.2%]	1.00^a^
24 Months				
Mobility	40.8% (20/49)	27.3% (6/22)	13.5% [− 9.6%, 36.7%]	0.27
Self-care	4.1% (2/49)	4.5% (1/22)	− 0.5% [− 10.8%, 9.9%]	1.00^a^
Usual activities	14.3% (7/49)	31.8% (7/22)	− 17.5% [−39.3%, 4.3%]	0.11^a^
Pain/discomfort	38.8% (19/49)	40.9% (9/22)	− 2.1% [− 26.8%, 22.5%]	0.86
Anxiety/depression	12.2% (6/49)	18.2% (4/22)	− 5.9% [− 24.5%, 12.6%]	0.49^a^

^a^*p*-values from 2-sided Fisher’s exact test

## Discussion

The 24-month results presented here support the efficacy and safety of DESs in Japanese patients. In this cohort, the 2-year reintervention rate for patients treated with Eluvia was approximately one-third that of patients treated with Zilver PTX and clinical improvements were sustained from 12 to 24 months. These results mirror those of the overall IMPERIAL RCT [10].

The literature on paclitaxel-containing endovascular therapy for Japanese patients with peripheral artery disease is not as extensive as that covering Caucasian populations. Favorable trends for maintaining patency and reducing reinterventions with drug-containing stents have been reported among Japanese patients [9, 11], but differences in lesion or patient characteristics or other study factors are potentially confounding and limit comparisons between studies. Ohki et al. [11] reported Kaplan–Meier estimates of freedom from TLR and primary patency of 96% and 80%, respectively, for the small cohort of Japanese patients treated with Zilver PTX in the Zilver PTX RCT (*n* = 25; mean lesion length 58.8 mm, 38.5% with occlusions). Patients in the large (*N* = 905) Zilver PTX Japan post-market surveillance study [9] had more complex lesions (mean lesion length 146 mm, 41.5% with occlusions), and the Kaplan–Meier estimates of freedom from CD-TLR and primary patency were 83.7% and 70.3%, respectively, at 2 years. Together with the findings for Eluvia DES treatment reported here, which demonstrated Kaplan–Meier freedom from TLR of 94.3% and primary patency of 88.5% at 24 months, these results suggest that paclitaxel-containing stents provide a clinical benefit to Japanese patients, with a more favorable trend observed for the polymer-based Eluvia DES.

Durability of DES outcomes is an important consideration given the landscape of other options for femoropopliteal treatment, such as drug-coated balloons. Iida et al. [15] reported 2-year primary patency of less than 80% among Japanese patients treated with drug-coated balloons who had lesion characteristics similar to those of the IMPERIAL patients (mean lesion length 91.5 mm, 16.2% with occlusions). The numerically better patency results reported here for patients treated with Eluvia suggest that DES may be appropriate to consider as a first choice, particularly for patients at greater risk of restenosis or more likely to require provisional stenting following balloon-based treatment [16].

The analyses reported here are limited by the small sample size and are unpowered for hypothesis testing. *P*-values for between-group comparisons are reported for reference. Although the cohort sample size is small, follow-up visit compliance was high with 97.3% of eligible patients (i.e., alive and not withdrawn) completing the 24-month visit to provide a comprehensive representation of the study cohort. Such exploratory analyses are valuable to probe generalizability of the global study results and examine whether patient outcomes may be differentially affected by practice- or population-based factors. Dedicated study of DES use among Japanese patients is required to investigate practice-related reasons for the outcomes observed. For example, intraprocedural use of intravascular ultrasound was not required or documented for purposes of the IMPERIAL study; however, it is commonly incorporated in Japanese endovascular practice and may have contributed to the excellent observed outcomes [17].

In conclusion, two-year results from the cohort of Japanese patients in IMPERIAL showed a numerically lower CD-TLR rate for Eluvia compared with Zilver PTX, and clinical outcome improvements sustained over time. This additional description of the Japanese cohort supports the applicability of the overall study conclusions to Japanese patients with clinical characteristics similar to those represented in the study.
